# Identification and characterization of genes frequently responsive to *Xanthomonas oryzae* pv. *oryzae* and *Magnaporthe oryzae* infections in rice

**DOI:** 10.1186/s12864-019-6438-y

**Published:** 2020-01-06

**Authors:** Weiwen Kong, Li Ding, Xue Xia

**Affiliations:** 1 School of Horticulture and Plant Protection, Yangzhou University, Yangzhou, 225009 Jiangsu China; 2 Joint International Research Laboratory of Agriculture and Agri-Product Safety of the Ministry of Education, Yangzhou University, Yangzhou, 225009 Jiangsu China

**Keywords:** Rice, *Xanthomonas oryzae* pv. *oryzae*, *Magnaporthe oryzae*, Transcriptome, Disease resistance, Disease susceptibility

## Abstract

**Background:**

Disease resistance is an important factor that impacts rice production. However, the mechanisms underlying rice disease resistance remain to be elucidated.

**Results:**

Here, we show that a robust set of genes has been defined in rice response to the infections of *Xanthomonas oryzae* pv. *oryzae* (*Xoo*) and *Magnaporthe oryzae* (*Mor*). We conducted a comprehensive analysis of the available microarray data from a variety of rice samples with inoculation of *Xoo* and *Mor*. A set of 12,932 genes was identified to be regulated by *Xoo* and another set of 2709 *Mor-*regulated genes was determined. GO enrichment analysis of the regulated genes by *Xoo* or *Mor* suggested mitochondrion may be an arena for the up-regulated genes and chloroplast be another for the down-regulated genes by *Xoo* or *Mor*. Cytokinin-related processes were most frequently repressed by *Xoo*, while processes relevant to jasmonic acid and abscisic acid were most frequently activated by *Xoo* and *Mor*. Among genes responsive to *Xoo* and *Mor*, defense responses and diverse signaling pathways were the most frequently enriched resistance mechanisms. InterPro annotation showed the zinc finger domain family, WRKY proteins, and Myb domain proteins were the most significant transcription factors regulated by *Xoo* and *Mor*. KEGG analysis demonstrated pathways including ‘phenylpropanoid biosynthesis’, ‘biosynthesis of antibiotics’, ‘phenylalanine metabolism’, and ‘biosynthesis of secondary metabolites’ were most frequently triggered by *Xoo* and *Mor*, whereas ‘circadian rhythm-plant’ was the most frequent pathway repressed by *Xoo* and *Mor*.

**Conclusions:**

The genes identified here represent a robust set of genes responsive to the infections of *Xoo* and *Mor*, which provides an overview of transcriptional reprogramming during rice defense against *Xoo* and *Mor* infections. Our study would be helpful in understanding the mechanisms of rice disease resistance.

## Background

Rice is one of the most staple food crops. During their entire growth, rice plants are capable of perceiving the invasion of a large number of micro-organisms, such as fungi, bacteria, and viruses. It has been established that plants are able to recognize and respond to various kinds of pathogens through their complex innate immunity systems [1, 2]. In the long-term struggle for survival, plants have evolved two lines of defense to pathogens, i.e., pathogen-associated molecular pattern (PAMP)-triggered immunity (PTI) and effector-triggered immunity (ETI) [3–5]. PTI could be activated by some extracellular, transmembrane receptors, also named pattern recognition receptors (PRRs) to suppress pathogen invasions. PRRs function in recognizing conserved PAMPs. The induction of ETI is the result of the recognition of pathogen avirulence effectors through disease resistance proteins of a host. During ETI response, hypersensitive reaction (HR), a specific programmed cell death (PCD), is often observed in plants. Many components have been demonstrated essential in the PTI and ETI processes in *Arabidopsis thaliana* [2, 6, 7].

Bacterial leaf blight is the most significant bacterial disease of rice. Its causal agent *Xanthomonas oryzae* pv. *oryzae* (*Xoo*), is a member of the gamma subdivision of the proteobacteria. Another popular rice disease is known as rice blast, which is caused by a filamentous ascomycete fungus, named *Magnaporthe oryzae* (*Mor*). Although disease resistance in rice has been extensively studied, there is still a little knowledge of the rice response to pathogen infections. It has been demonstrated that the invasions of *Xoo* and *Mor* on rice plants are mainly mediated through altering rice gene expression at the transcriptional level [8–11]. Hence, uncovering the transcriptional changes of rice genes during the infections of *Xoo* and *Mor* is of particular significance.

In rice plants, PTI and ETI were observed in response to *Xoo* [12] and *Mor* infection [13, 14]. Extensive researches have revealed that some phytohormones, e.g., jasmonic acid (JA), abscisic acid (ABA), salicylic acid (SA), and ethylene (ET), are important in the rice responses to *Xoo* [15–20] and *Mor* infections [21–23].

Up to date, plenty of microarray data of rice infected by *Xoo* and *Mor* have been produced, and these data give an opportunity to elucidate the mechanisms of rice response to the infections of the two pathogens. Previous microarray-based studies, however, focused on only a limited of samples. Although numerous rice genes responsive to *Xoo* and *Mor* infections have been identified, which led to insights into the rice resistance/susceptibility mechanisms, similar or specific results were usually obtained in different studies for the sake of specific experimental conditions.

The aim of this study is to determine a robust set of rice genes in response to *Xoo* and *Mor* infections, defining genes that are frequently regulated in diverse conditions through analysis of the publicly available rice microarray data sets associated with the infections of *Xoo* and *Mor*. To find out which mechanisms may be more common in rice response to the infections by these two pathogens, the gene sets determined were next analyzed for enrichment of Gene Ontology (GO) terms and KEGG (Kyoto Encyclopedia of Genes and Genomes) pathways. Furthermore, the distributions of the significant enrichment of the GO and KEGG as well as the InterPro annotation were investigated.

## Results

### Identification of genes responsive to *Xoo* and *Mor* infections in the rice microarray data

We employed 69 pairs (including control and treatment) of microarray samples (consisting of 51 pairs of *Xoo*-infected samples and 18 pairs of *Mor*-infected samples) from 15 series of experiments to query *Xoo-* and *Mor-*induced gene expression changes, all of which were conducted by the use of the Affymetrix rice whole-genome arrays platform (GPL2025) (Additional file 4: Table S1). The data from the same platform were retrieved for the analysis to avoid the variance of different platforms, and the GEO2R tool was used to process all the samples uniformly to eliminate the technical variance of data transformation. Moreover, poor quality arrays with no match or matching multiple loci were discarded. Further, we only considered the samples with no less than 989 differentially expressed genes (DEGs) (*P* ≤ 0.05). In the end, we identified the DEGs from 29 pairs of *Xoo*-infected samples and 6 pairs of *Mor*-infected samples (Additional file 5: Table S2). The number of DEGs in these samples varied from 989 to 9769 genes (Additional file 5: Table S2). Totally, 12,932 DEGs (occurring at least three pairs of array samples) were identified in the *Xoo*-infected rice microarray data (Table 1)*.* Of the DEGs, 7452 genes were up-regulated and 5480 genes were down-regulated (Table 1). Also, 2709 DEGs (occurring at least three pairs of array samples) were identified in the *Mor*-infected rice microarray data (Table 1)*.* Out of these genes, 1615 were up-regulated and 1094 were down-regulated by *Mor* (Table 1).

**Table 1:** The number of differentially expressed genes identified in the rice microarray data with inoculation of *Xoo* and *Mor*

Number of sample	Number of up-reg gene	Number of down-reg gene
Inoculation with *Xoo*		
22	1	0
21	1	1
20	2	1
19	4	3
18	9	9
17	20	24
16	40	38
15	85	74
14	180	156
13	318	264
12	561	422
11	850	644
10	1209	925
9	1598	1256
8	2118	1657
7	2759	2125
6	3562	2702
5	4567	3411
4	5969	4297
3	7452	5480
2	9360	7445
1	12,062	10,372
Inoculation with *Mor*		
6	15	0
5	179	10
4	640	145
3	1615	1094
2	3210	3830
1	6928	8389

*up-reg* up-regulated, *down-reg* down-regulated

**Fig. 1:**
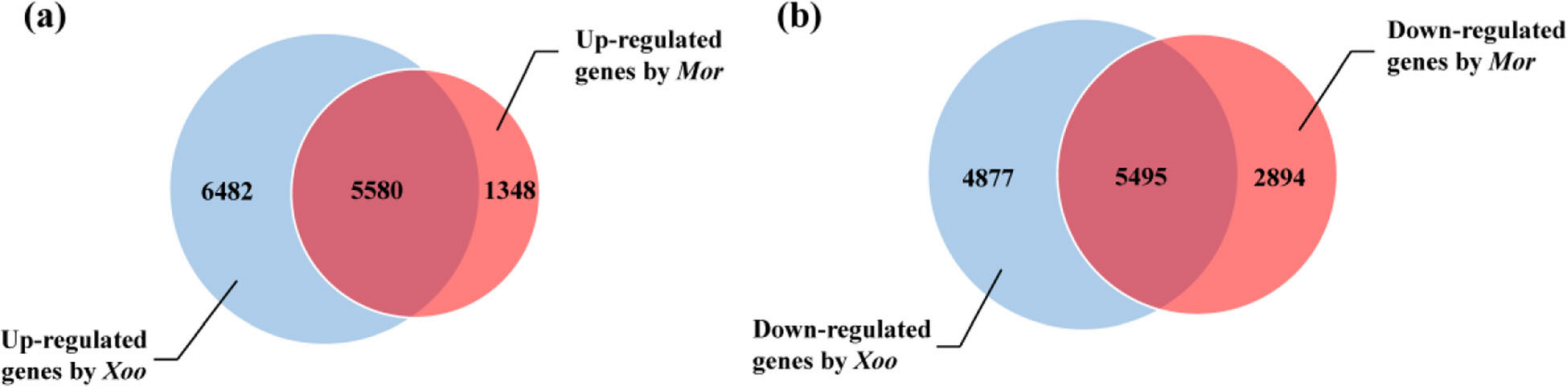
Number of unique and common differentially expressed genes (DEGs) induced by *Xoo* and *Mor* in rice. **a** Up-regulated genes; **b** Down-regulated genes

Comparing the two groups of the identified DEGs, we found that 11,075 genes were expressed differentially in common between *Xoo-* and *Mor-* infected rice array samples, with 5580 DEGs being up-regulated and 5495 DEGs being down-regulated (Fig. 1). If only considering the DEGs that were present in at least three pairs of samples infected by *Xoo* or in at least two pairs of samples infected by *Mor*, it could be found that 3831 DEGs were shared by *Xoo-* and *Mor-* infected array samples, with 2140 DEGs being up-regulated and 1691 DEGs being down-regulated (Additional file 1: Figure S1).

In a previous study, we showed that 882 rice genes contain pathogen-inducible *cis*-regulatory elements (PICEs) in their promoter regions [24]. Here we found that 389 DEGs contain the PICEs in their promoters (Additional file 3: Table S3). There is a 3.51% (389/11075) overlap or a 44.1% (389/882) overlap between the DEGs and the genes with PICEs in the promoters. And among the above 3831 DEGs, 304 genes were found to be overlapped with the prior set of genes carrying the PICEs in their promoters, which account for 7.93% of the DEGs (Additional file 7: Table S4). It seems that the PICEs in the promoters make genes accessible to be frequently regulated by pathogens.

### GO enrichment analysis of the DEGs in the *Xoo-* and *Mor-*infected rice samples

**Fig. 2:**
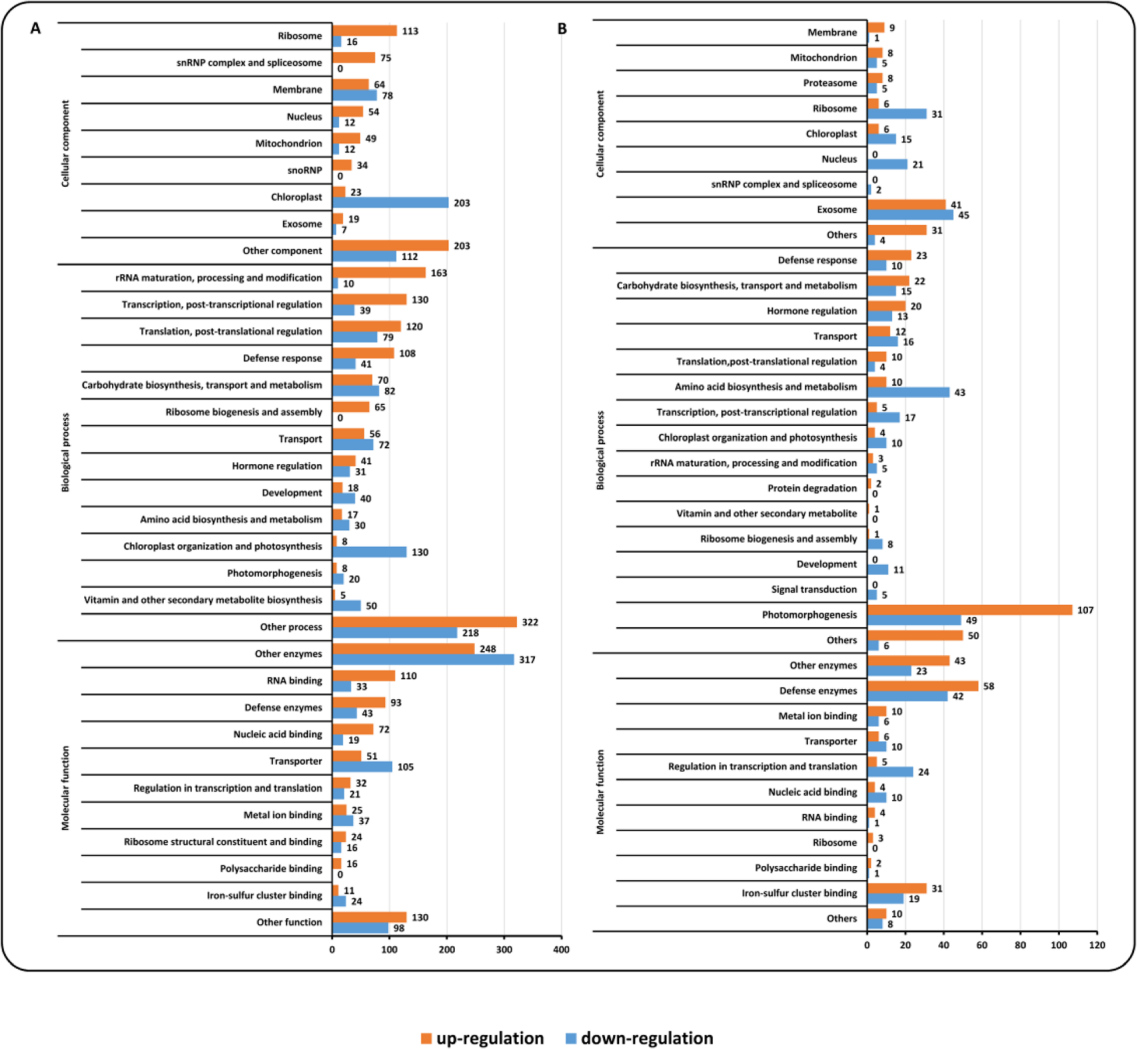
The distribution of the gene ontology annotation (GO) for the induced genes in the rice microarray data. **a** GO for the induced genes by *Xoo*; **b** GO for the induced genes by *Mor*

GO enrichment analysis provides some detailed information on the potential functions of genes. However, the existing GO terms are too disorganized to succinctly describe the functional information of a large set of genes. Here, combining the routine GO terms, we tentatively use more broad terms to describe the GO enrichment results of the DEGs in the *Xoo-* and *Mor-*infected rice samples. GO enrichment analysis revealed that ribosome, snRNP complex and spliceosome, and diverse membranes were the most frequent cellular components in the up-regulated genes by *Xoo* (Fig. 2a and Additional file 8: Table S5). In contrast, chloroplast, membrane, and ribosome were the most frequent cellular components in the down-regulated genes by *Xoo* (Fig. 2a and Additional file 9: Table S6).

GO enrichment results also demonstrated that the most frequent biological processes were: (1) rRNA maturation, processing, and modification; (2) transcription, post-transcription regulation; (3) translation, post-translation regulation, and (4) defense response in the up-regulated genes by *Xoo* (Fig. 2a and Additional file 10: Table S7). In the down-regulated genes by *Xoo*, the most frequently enriched biological processes were: (1) chloroplast organization and photosynthesis; (2) carbohydrate biosynthesis, transport and metabolism; (3) translation, post-translational regulation; and (4) transport (Fig. 2a and Additional file 11: Table S8).

Analysis on the molecular functions of the genes induced by *Xoo* showed that RNA binding, defense enzymes and other enzymes were significantly enriched in the up-regulated genes (Fig. 2a and Additional file 12: Table S9). By contrast, defense enzymes and other enzymes, as well as transporters were the frequently enriched molecular function categories in the down-regulated genes by *Xoo* (Fig. 2a and Additional file 13: Table S10).

In the up-regulated genes by *Mor*, the analysis presented that the most frequently enriched cellular components were membrane, mitochondrion, proteasome, and ribosome (Fig. 2b and Additional file 14: Table S11); whereas the most frequently enriched cellular components were chloroplast, snRNP complex and spliceosome, and nucleus in the genes down-regulated by *Mor* (Fig. 2b and Additional file 15: Table S12).

On the biological processes that the induced genes by *Mor* involved in, enrichment analysis indicated that defense response, carbohydrate biosynthesis, transport and metabolism, and hormone regulation were the most frequent processes present in the up-regulated genes (Fig. 2b and Additional file 16: Table S13); while transcription, post-transcriptional regulation, chloroplast and photosynthesis, and translation, post-translational regulation were the most frequent processes occurring in the down-regulated genes (Fig. 2b and Additional file 17: Table S14).

Enrichment on the GO molecular functions of the regulated genes by *Mor* showed that defense enzymes, other enzymes, metal ion binding, and transporter were the most frequent categories in the up-regulated genes (Fig. 2b and Additional file 18: Table S15); while in the down-regulated genes, nucleic acid binding, defense enzymes and other enzymes were included in the most frequent molecular functions (Fig. 2b and Additional file 19: Table S16).

**Fig. 3:**
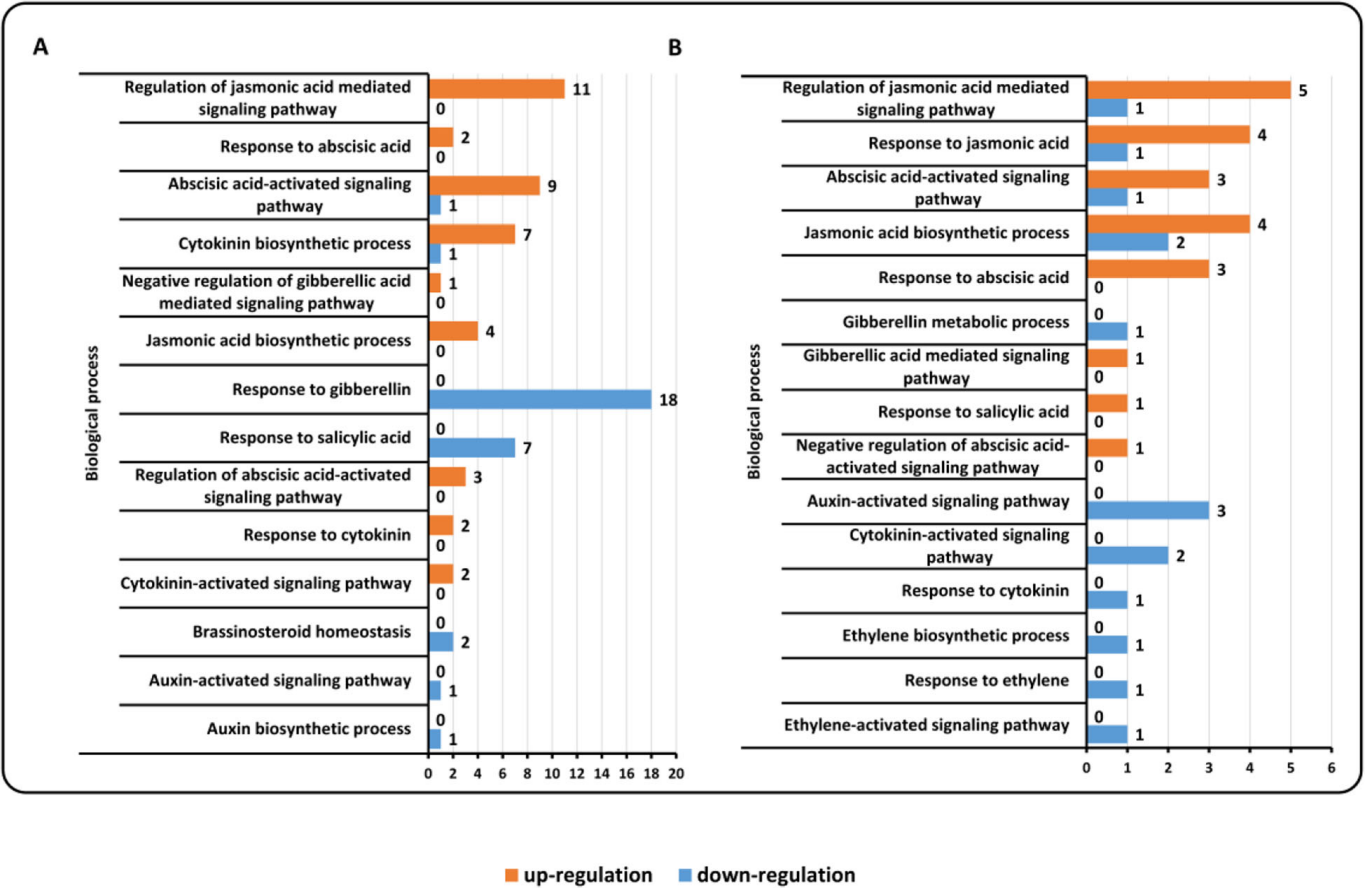
The distribution of the biological process (BP) relative to hormone regulation for the induced genes in the rice microarray data. **a** BP for the induced genes by *Xoo*; **b** BP for the induced genes by *Mor*

It has been established that most hormones are important in regulating rice disease resistance. Thus, we investigated the biological processes related to diverse hormones, which were activated or repressed after the infections of *Xoo* and *Mor*. We observed that some biological processes relative to hormones included the regulation of hormone-mediated signaling pathways, hormone biosynthetic/metabolic processes, and response to hormone (Fig. 3). Among the hormones, jasmonic acid and abscisic acid were prominent for the processes related to them were most frequently activated after infections by *Xoo* and *Mor* (Fig. 3). It is worth noting that the processes related to cytokinin (GO:0009735 and GO:0009736) were most frequently repressed after *Xoo* infection (Fig. 3a). In samples infected by *Mor*, the processes relative to diverse hormones consisting of abscisic acid, auxin, cytokinin, jasmonic acid, and ethylene were evenly repressed (Fig. 3b).

Comparison of the most frequently observed GO enrichment results of the induced genes by *Xoo* and *Mor* revealed some interesting phenomena. Ribosome, membrane, mitochondria, and chloroplast were frequently observed, suggesting they were important cellular components where many genes were induced during the infections of *Xoo* or *Mor*. However, the occurring frequencies were different between the up- and down-regulated patterns (Fig. 5). For example, the mitochondrion was a cellular component where the occurring frequency was higher in the up-regulated genes than the down-regulated genes by *Xoo* and *Mor* (Fig. 2 and Fig. 5). Conversely, the occurring frequency of the chloroplast was higher in the down-regulated genes than the up-regulated genes by both of the pathogens (Fig. 2 and Fig. 5). Therefore, when rice plants are subject to *Xoo* and *Mor* infections, mitochondria and chloroplast will turn into two important arenas, with the former being the one for up-regulated genes and the latter being another for down-regulated genes. Also, among the biological processes, defense response and hormone regulation were observed to be commonly and frequently activated by these two pathogens through up-regulating some related genes; and chloroplast organization and photosynthesis, development, and photomorphogenesis were commonly and frequently repressed by the two pathogens through down-regulating the relevant genes (Fig. 2 and Fig. 5). Similarly, the molecular functions including defense enzymes, ribosome structural constituent and binding, and polysaccharide binding were commonly and frequently observed in the up-regulated genes by the two pathogens (Fig. 2 and Fig. 5).

In addition, some frequent GO enrichment results were observed to be specific (Fig. 5). For example, ‘snRNP complex and spliceosome’ was only frequently occurring in the genes up-regulated by *Xoo* and down-regulated by *Mor*; snoRNP was only frequently occurring in the up-regulated genes by *Xoo* (Fig. 2 and Fig. 5). The genes involved in ribosome biogenesis and assembly process were only frequently observed among the genes with an up-regulated pattern of expression induced by *Xoo* and *Mor* (Fig. 2 and Fig. 5). And the signal transduction process was only observed among the genes down-regulated by *Mor* (Fig. 2b and Fig. 5). The genes with polysaccharide binding function were exclusively identified in those with an up-regulated pattern of expression induced by the two pathogens (Fig. 2 and Fig. 5). Hence, some results obtained from the analysis of microarray data infected by *Mor* further supported that from the analysis of data infected by *Xoo*; on the other hand, comparison of the data infected by *Mor* to that by *Xoo* indicated that rice plants can use different mechanisms in response to their infections.

### Enriched InterPro annotation of the DEGs in rice induced by *Xoo* and *Mor* infections

To further explore the possible functions of the DEGs in rice induced by *Xoo* and *Mor* infections, InterPro annotation enrichment analysis was conducted. The most frequent and significant InterPro annotations associated with the up-regulated DEGs by *Xoo* were diverse kinds of enzymes, e.g., dehydrogenase, synthase/synthetase, transferase, kinase, and glycoside hydrolase, followed by ribosomal proteins, translation proteins, and transcription factors; while among the down-regulated DEGs by *Xoo*, the most frequent and significant InterPro annotations were diverse enzymes including transferase, synthase/synthetase, reductase, dehydrogenase, and peptidase, etc., followed by transporter, transcription factors and ribosomal proteins (Table 2, Additional file 20: Table S17 and Additional file 21: S18).

**Table 2:** The distribution of the enriched INTERPRO annotations of the genes induced by *Xoo* in the rice microarray data

Annotation	Frequency in the analyzed samples	
	Up-regulation	Down-regulation
Enzyme	1087	737
Dehydrogenase	149	56
Synthase/synthetase	123	124
Transferase	122	137
Kinase	87	32
Glycoside hydrolase	79	28
ATPase	60	15
Helicase	48	10
Phosphatase	48	13
Peptidase	38	36
Reductase	23	72
Peroxidase	20	1
Lipoxygenase	16	0
Other enzymes	274	213
Ribosomal protein	498	44
Translation protein	88	3
Transcription factor	68	63
Transporter	62	128
WD40	39	0
Tubulin	32	3
Heat shock protein	26	8
Others	972	842

In the up-regulated DEGs by *Mor*, the analysis showed that diverse kinds of enzymes (e.g., dehydrogenase, kinase, transferase, hydrolase, and synthase/synthetase), transporters, and transcription factors were the top three enriched InterPro annotations; and in the down-regulated DEGs by *Mor*, it was demonstrated that various enzymes including kinase, transferase, synthase/synthetase, and so on, were annotated to be the most frequent products associated with the DEGs, followed by transcription factors, heat shock proteins and WD40 domain proteins (Table 3, Additional file 22: Table S19 and Additional file 23: Table S20.). Therefore, a number of enzymes were induced in rice response to *Xoo* and *Mor* infections, with some being up-regulated and others down-regulated. Among the enzymes, kinase, transferase, and synthase/synthetase were frequently activated or repressed during the rice response to *Xoo* and *Mor* infections. According to the above GO molecular function analysis of the DEGs, some activated enzymes belong to defense enzymes.

**Table 3:** The distribution of the enriched INTERPRO annotations of the up-regulated genes induced by *Mor* in the rice microarray data

Annotation	Frequency in the analyzed samples	
	Up-regulation	Down-regulation
Enzyme	382	143
Dehydrogenase	52	9
Kinase	50	18
Transferase	47	13
Hydrolase	34	8
Synthase/synthetase	33	11
Peptidase	18	9
Reductase	15	2
ATPase	12	3
Lipoxygenase	12	0
Helicase	8	10
Phosphatase	7	8
Peroxidase	1	1
Other enzymes	93	51
Transporter	34	5
Transcription factor	15	69
Ribosomal protein	8	7
Heat shock protein	5	8
Translation protein	3	3
WD40	0	8
Others	209	268

### Disease resistance/susceptibility-related (DRR/DSR) genes associated with *Xoo* and *Mor* infections in rice

To identify the DRR/DSR genes, we performed a more detailed InterPro analysis on the *Xoo*- and *Mor*-regulated genes, combining with literature mining. Among the up-regulated genes by *Xoo*, the analysis indicated that genes from 106 InterPro annotated items were identified as DRR/DSR genes and the most frequently up-regulated genes by *Xoo* encode NAD(P)-binding domain proteins (IPR016040), which involve in reactive oxygen species (ROS) and SA signaling (Additional file 24: Table S21). Genes encoding chaperonin Cpn60/TCP-1 (IPR002423) and GroEL-like apical domain proteins (IPR027409) were also frequently activated, which are related to PCD and defense response, respectively (Additional file 24: Table S21). Among the down-regulated genes by *Xoo*, the analysis showed that genes from 75 InterPro items were identified as DRR/DSR genes (Additional file 25: Table S22). Three groups of genes were most frequently down-regulated by *Xoo*, and they encode NAD(P)-binding domain proteins (IPR016040) (functioning in ROS and SA signaling), major facilitator superfamily (IPR020846 and IPR011701) (acting as defense proteins), and NAF/FISL domain proteins (IPR004041 and IPR018451) (involved in PTI), respectively (Additional file 25: Table S22).

Among genes responsive to *Mor*, the results demonstrated that genes from 39 InterPro annotated items were identified to be DRR/DSR genes, with up-regulated expression pattern (Additional file 26: Table S23). Interestingly, the most frequently activated genes by *Mor* also encode NAD(P)-binding domain proteins (IPR016040), and function in ROS and SA signaling (Additional file 26: Table S23). Among the down-regulated genes by *Mor*, the putative DRR/DSR genes were confirmed to be distributed in 19 InterPro annotated items (Additional file 27: Table S24). Three groups of genes, which encode PB1 domain (Phox/Bem1p) proteins (IPR000270) (involved in defense response), DnaJ domain proteins (IPR001623) (implicated in cell death), and CCT domain proteins (IPR010402) (related to defense response), respectively, were most frequently repressed by *Mor* infection (Additional file 27: Table S24).

**Fig. 4:**
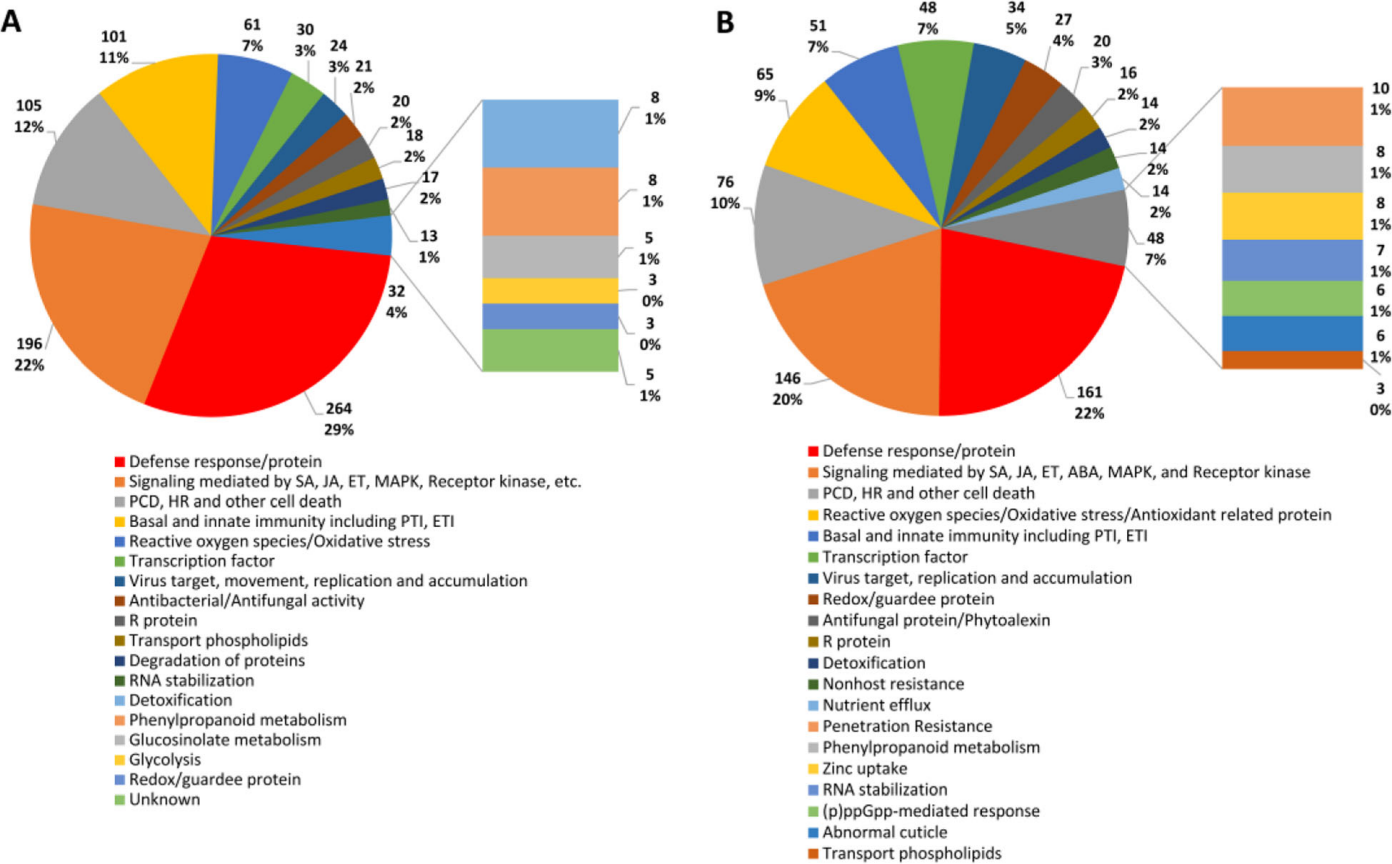
Analysis on disease resistance mechanisms of the up-regulated DRR/DSR proteins in rice. **a** mechanism for the up-regulated proteins by *Xoo*; **b** mechanism for the up-regulated proteins by *Mor*

We further investigated the mechanisms on disease resistance of the identified DRR/DSR genes in rice response to *Xoo* and *Mor* infections through consulting a great number of papers. The results showed that various disease resistance mechanisms were conferred by the up-regulated genes by *Xoo* (Fig. 4a). For example, 29% (264) of the up-regulated genes by *Xoo* played roles during defense responses or as defense proteins. One hundred ninety six genes (22%) were probably involved in diverse signaling pathways, including SA, JA, ET, MAPK, receptor kinase and so on, among the up-regulated genes by *Xoo*. 12% (105) of the up-regulated genes by *Xoo* were associated with PCD, HR or other cell death. A group of 101 up-regulated genes by *Xoo* (11%) was related to basal and innate immunity including PTI, ETI. 7% (61) were implicated in ROS/oxidative stress, and 3% (30) were found as transcription factors, among the up-regulated genes by *Xoo* (Fig. 4a). A similar result was observed among the down-regulated genes by *Xoo* (Fig. 4b). For instance, 22% (161) of the down-regulated genes by *Xoo*, the greatest group of genes, played a part in defense responses or as defense proteins. A group of 146 genes (20%) was related to diverse signaling pathways mediated by SA, JA, ET, ABA, MAPK, and receptor kinase, among the down-regulated genes by *Xoo*. 10% (76) of the down-regulated genes by *Xoo* were in association with PCD, HR and other cell death. 9% (65) were involved in ROS, oxidative stress or antioxidant related protein. Another group of 51 down-regulated genes by *Xoo* (7%) was implicated in basal and innate immunity including PTI, ETI. In addition, 48 down-regulated genes by *Xoo* (6%) were present, which encoded transcription factors (Fig. 4b).

Analysis of the genes regulated by *Mor* revealed some disease resistance mechanisms (Additional file 1: Figure S1). Defense responses (49 genes, 27%) were observed to be most frequently induced in rice after infection by *Mor*. A group of 32 genes (18%) activated by *Mor* was implicated in signaling mediated by SA, ET, MAPK, etc. Some induced genes encoded antifungal proteins or were related to the synthesis of phytoalexin. Induced genes involving in PCD, HR and other cell death, and genes related to ROS accounted for 10% (19) and 8% (15), respectively (Additional file 2: Figure S2). Among the down-regulated genes by *Mor*, 54% (27) encoded transcription factors; ten genes (20%) were associated with cell death; four genes (8%) were present in defense responses; additionally, several genes were related to auxin signaling, ethylene signaling, and NPR1 degradation, respectively (Additional file 3: Figure S3).

### Top regulated genes after *Xoo* and *Mor* infections

We next determined the top regulated genes by *Xoo* and *Mor* in rice. We found that nine genes, which were occurring in at least 18 pairs of samples, were up-regulated after *Xoo* infection (Table 4). *Os04g0650800*, which encodes the D-3-phosphoglycerate dehydrogenase 3 in chloroplast, was found to be most frequently up-regulated, with occurring in 22 pairs of *Xoo*-infected samples (Table 4). Four of the up-regulated genes are involved in defense responses (*Os03g0235000*, *Os09g0491772*, *Os08g0508800*, and *Os09g0484200*) (Table 4). Another nine genes were observed to be frequently down-regulated by *Xoo*, with happening in at least 18 pairs of samples (Table 5). Among the genes, one encodes the cinnamoyl-CoA reductase 1 (*Os09g0491820*), and was most frequently down-regulated by *Xoo*, with occurring in 21 pairs of samples; two genes (*Os05g0204600* and *Os06g0571800*) encode different transcription factors (Table 5).

**Table 4:** Top 20 up-regulated genes in rice after Xoo infection

Gene ID	Annotation	Number of sample infected by *Xoo* (*n* = 29)
*Os04g0650800*	D-3-phosphoglycerate dehydrogenase 3, chloroplastic	22
*Os03g0235000*	peroxidase A2	20
*Os08g0127100*	lysine histidine transporter 1	19
*Os10g0444700*	probable inorganic phosphate transporter 1–8	19
*Os09g0491772*	heat shock 70 kDa protein, mitochondrial	18
*Os08g0508800*	lipoxygenase 7, chloroplastic	18
*Os02g0720600*	protein ASPARTIC PROTEASE IN GUARD CELL 1	18
*Os09g0484200*	cytochrome c1	18
*Os06g0150400*	uncharacterized LOC4340145	18
*Os06g0547400*	peroxidase P7	17
*Os09g0412400*	uncharacterized LOC4347040	17
*Os12g0131100*	glutamine--fructose-6-phosphate aminotransferase [isomerizing] 2	17
*Os01g0830700*	protein trichome birefringence-like 28	17
*Os02g0627100*	phenylalanine ammonia-lyase	17
*Os03g0213100*	protein transport protein Sec61 subunit alpha	17
*Os11g0684000*	transcription factor MYB108	17
*Os07g0550600*	benzyl alcohol O-benzoyltransferase	17
*Os01g0839300*	50S ribosomal protein L17	17
*Os06g0116600*	Proteinase inhibitor, propeptide domain containing protein	17
*Os01g0217500*	protein DJ-1 homolog B	17

**Table 5:** Top 10 down-regulated genes in rice after *Xoo* infection

Gene ID	Annotation	Number of sample infected by Xoo *(n = 29)*
*Os09g0491820*	cinnamoyl-CoA reductase 1	21
*Os05g0204600*	B-box zinc finger protein 22	19
*Os04g0532400*	salutaridine reductase	19
*Os12g0124000*	nuclear envelope pore membrane protein POM 121	18
*Os01g0702000*	bifunctional nuclease 1	18
*Os06g0571800*	putative GATA transcription factor 22	18
*Os12g0529900*	myosin heavy chain, striated muscle	18
*Os08g0480000*	protein DETOXIFICATION 27	18
*Os04g0538100*	elongation factor G-1, chloroplastic	18
*Os01g0763700*	glycerophosphodiester phosphodiesterase GDPD6	17

In *Mor*-infected samples, 16 genes were observed to be up-regulated in all the six pairs of samples analyzed (Table 6). Among these genes, five belong to defense genes (*Os01g0713200*, *Os01g0963000*, *Os02g0569900*, *Os02g0570700*, and *Os10g0542900*), one encodes a WRKY transcription factor (*Os01g0584900*), and another gene (*Os04g0647900*) encodes an LRR receptor-like serine/threonine-protein kinase (Table 6). In the samples, ten genes were found to be down-regulated, with occurring in five pairs of samples (Table 7). Out of the genes, *Os01g0719100* and *Os03g0607700* encode two different zinc finger proteins, and *Os09g0536400* encodes a defense protein (thaumatin-like protein 1b) (Table 7).

**Table 6:** Top 16 up-regulated genes in rice after *Mor* infection

Gene ID	Annotation	Number of sample infected by *Xoo* (*n* = 6)
*Os01g0389200*	uncharacterized LOC4325227	6
*Os01g0584900*	WRKY transcription factor SUSIBA2	6
*Os01g0695800*	ABC transporter B family member 11	6
*Os01g0713200*	glucan endo-1,3-beta-glucosidase GII	6
*Os01g0963000*	cationic peroxidase SPC4	6
*Os02g0569900*	Cytochrome P450 family protein	6
*Os02g0570700*	Cytochrome P450 family protein	6
*Os04g0647900*	LRR receptor-like serine/threonine-protein kinase GSO1	6
*Os06g0128800*	C2 calcium/lipid-binding domain, CaLB domain containing protein	6
*Os06g0226950*	Fatty acid hydroxylase domain containing protein	6
*Os06g0569500*	ent-sandaracopimaradiene 3-hydroxylase	6
*Os08g0137800*	Cupredoxin domain containing protein	6
*Os08g0189900*	germin-like protein 8–11	6
*Os08g0190100*	germin-like protein 8–11	6
*Os10g0542900*	chitinase 8	6
*Os12g0555000*	Similar to Probenazole-inducible protein PBZ1	6

**Table 7:** Top 10 down-regulated genes in rice after *Mor* infection

Gene ID	Annotation	Number of sample infected by *Xoo* (*n* = 6)
*Os07g0210000*	exocyst complex component EXO70B1	5
*Os01g0719100*	RING zinc-finger protein, Stomata opening	5
*Os02g0732900*	APO protein 2, chloroplastic	5
*Os01g0933600*	uncharacterized LOC9268621	5
*Os09g0479100*	F-box domain, cyclin-like domain containing protein	5
*Os10g0465000*	WD repeat-containing protein 26	5
*Os07g0584900*	U-box domain-containing protein 4	5
*Os03g0607700*	Zinc finger, C2H2-like domain containing protein.	5
*Os08g0176100*	phosphopantothenate--cysteine ligase 2	5
*Os09g0536400*	thaumatin-like protein 1b	5

### Frequently observed transcription factors induced by *Xoo* and *Mor* in rice

Transcription factors are pivotal components that regulate gene expression. Thus, we identified the differentially expressed transcription factors in the *Xoo*- and *Mor*-infected microarray data. InterPro annotation indicated that diverse zinc finger proteins were the most frequently observed among the up-regulated transcription factors in the *Xoo*-infected samples, and heat shock factors (IPR000232) were the second most frequently observed, followed by WRKY (IPR003657) and basic-leucine zipper domain-containing proteins (IPR004827) (Additional file 8: Table S5). In the samples, zinc finger proteins were also the most frequently observed among the down-regulated transcription factors, whereas heat shock factors (IPR000232) and WRKY (IPR003657) were not observed (Additional file 28: Table S25). Instead, Myb domain proteins were the second most frequently observed in the down-regulated transcription factors in the *Xoo*-infected samples (Additional file 28: Table S25).

**Fig. 5:**
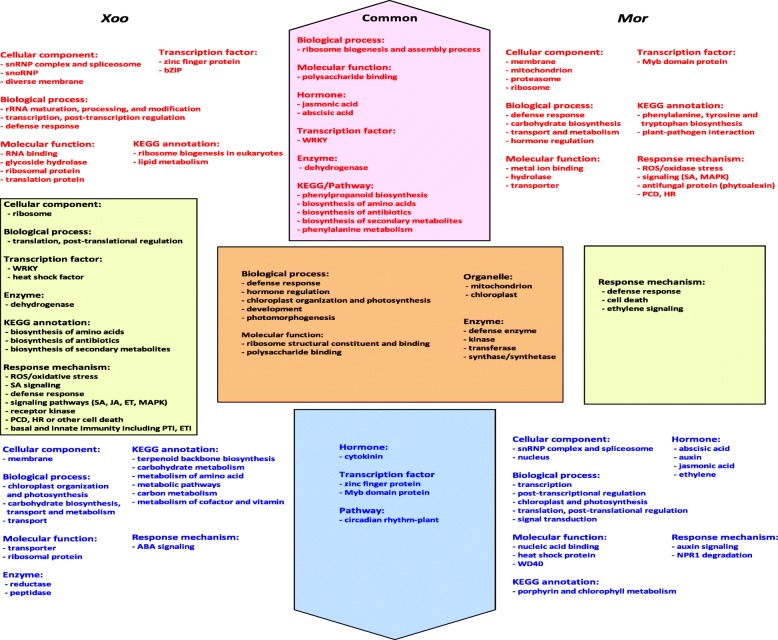
Diagram of frequent rice terms in response to the infections of *Xoo* and *Mor*. Terms in pink up-pentagon show those that were commonly up-regulated in rice response to *Xoo* and *Mor* infections, and the terms in each side of the pink up-pentagon show those that were specifically up-regulated in response to the infection by one of the two pathogens. Similarly the terms in light blue down-pentagon and those in both the sides of the down-pentagon demonstrate the terms that were down-regulated in the infections by both pathogens and specific to one infection respectively. Terms in light yellow rectangles were found both in up- and down-regulated gene sets in response to one infection respectively. Terms in brown rectangle were found both in up- and down-regulated gene sets

Of the up-regulated transcription factors in the *Mor*-infected samples, the WRKY proteins (IPR003657) were the maximum of the induced transcription factors, followed by the Myb domain factors (IPR006447, IPR017930) (Additional file 29: Table S26). Among the down-regulated transcription factors in these samples, various kinds of zinc finger domain proteins were also the most frequently induced transcription factors, and the Myb domain proteins (IPR006447, IPR017930, and IPR001005) were the second frequently induced (Additional file 29: Table S26). Therefore, the zinc finger domain family, WRKY proteins, and Myb domain proteins were the most significant proteins among the differentially expressed transcription factors in most of the *Xoo*- and *Mor*-infected samples (Fig. 5).

### KEGG enrichment analysis of the DEGs induced by *Xoo* and *Mor* in rice

To search the enriched pathways potentially targeted by the DEGs in rice induced by *Xoo* and *Mor* infections, KEGG annotation was performed. The analysis indicated that genes involved in the following pathways were most frequently enriched in the up-regulated genes by *Xoo*: ‘phenylpropanoid biosynthesis’ (osa00940), ‘biosynthesis of amino acids’ (osa01230), ‘biosynthesis of antibiotics’ (osa01130), ‘ribosome biogenesis in eukaryotes’ (osa03008), and ‘biosynthesis of secondary metabolites’ (osa01110) pathways (Additional file 30: Table S27). In the down-regulated genes by *Xoo*, the most frequent pathways targeted were ‘biosynthesis of secondary metabolites’ (osa01110), ‘metabolic pathways’ (osa01100), ‘biosynthesis of antibiotics’ (osa01130), and ‘carbon metabolism’ (osa01200) (Additional file 31: Table S28).

To better outline the enrichment results of the KEGG targeted by the DEGs in rice induced by *Xoo* and *Mor*, all the pathways enriched were divided into eight categories (Table 8, Table 10). As shown in Table 8, disease resistance related pathways, carbohydrate metabolism, and genetic information processing were the top three frequently enriched categories in the up-regulated genes by *Xoo*; and in the down-regulated genes by *Xoo*, carbohydrate metabolism, disease resistance related pathways, and biosynthesis and metabolism of amino acids were observed to be the most frequently enriched pathways. Comparison of the pathways targeted by the genes induced by *Xoo* indicates that the pathways related to genetic information processing and lipid metabolism were more frequently enriched in the up-regulated genes, while the pathways involving metabolism of cofactors and vitamins were more frequently enriched in the down-regulated genes (Table 8).

**Table 8:** The distribution of the enriched KEGG of the genes induced by *Xoo* in the rice microarray data

Pathway category	Frequency in the analyzed samples	
	Up-regulation	Down-regulation
Disease resistance related pathways	101	70
Carbohydrate metabolism	82	76
Genetic Information Processing	79	14
Biosynthesis and metabolism of amino acids	75	69
Lipid metabolism	30	10
Biosynthesis of secondary metabolites	22	35
Metabolism of cofactors and vitamins	4	43
Other pathways	68	124

Among the disease resistance related pathways, ‘phenylpropanoid biosynthesis’ (osa00940), ‘biosynthesis of antibiotics’ (osa01130), and ‘phenylalanine metabolism’ (osa00360) were the most frequent pathways activated by *Xoo*; however, ‘biosynthesis of antibiotics’ (osa01130), ‘circadian rhythm-plant’ (osa04712), and ‘terpenoid backbone biosynthesis’ (osa00900) were the most frequent pathways repressed by *Xoo* (Table 9).

**Table 9:** The distribution of the enriched KEGG pathways related to disease resistance of the genes induced by *Xoo* in the rice microarray data. Up-Reg: Up-regulation; Down-Reg: Down-regulation

Disease resistance related pathways	Reference	Number of sample	
		Up-Reg	Down-Reg
osa00940:Phenylpropanoid biosynthesis	[25–28]	16	3
osa01130:Biosynthesis of antibiotics		15	18
osa00360:Phenylalanine metabolism	[29–31]	14	0
osa00400:Phenylalanine, tyrosine and tryptophan biosynthesis	[32]	11	0
osa04075:Plant hormone signal transduction	[21, 23, 33–37]	10	5
osa00945:Stilbenoid, diarylheptanoid and gingerol biosynthesis	[38–40]	9	0
osa03450:Non-homologous end-joining	[41]	8	0
osa04712:Circadian rhythm - plant	[42–44]	7	16
osa04626:Plant-pathogen interaction		6	2
osa00941:Flavonoid biosynthesis	[45]	5	3
osa00900:Terpenoid backbone biosynthesis	[46]	0	12
osa00908:Zeatin biosynthesis	[47]	0	7
osa00480:Glutathione metabolism	[48]	0	2
osa00909:Sesquiterpenoid and triterpenoid biosynthesis	[49, 50]	0	2

We next analyzed the KEGG enrichment of the genes induced by *Mor*. This analysis led to a similar KEGG enrichment of the up-regulated genes by *Mor* to that of the genes activated by *Xoo*. As listed in Additional file 32: Table S29, ‘biosynthesis of secondary metabolites’ (osa01110), ‘biosynthesis of amino acids’ (osa01230), ‘phenylpropanoid biosynthesis’ (osa00940), and ‘biosynthesis of antibiotics’ (osa01130) were also frequently enriched in *Mor*-up-regulated genes. However, in the *Mor*-down-regulated genes, the frequent KEGG enrichment annotations were ‘plant hormone signal transduction’ (osa04075), ‘circadian rhythm-plant’ (osa04712), and ‘porphyrin and chlorophyll metabolism’ (osa00860) (Additional file 33: Table S30). Broad KEGG enrichment results indicated that disease resistance related pathways, biosynthesis and metabolism of amino acids, and carbohydrate metabolism categories were most frequently enriched in the up-regulated genes by *Mor*; while in the genes repressed by *Mor*, the most frequent enrichment categories of KEGG were disease resistance related pathways, genetic information processing, and biosynthesis and metabolism of amino acids (Table 10).

**Table 10:** The distribution of the enriched KEGG of the genes induced by *Mor* in the rice microarray data

Pathway category	Frequency in the analyzed samples	
	Up-regulation	Down-regulation
Disease resistance related pathways	38	17
Biosynthesis and metabolism of amino acids	27	9
Carbohydrate metabolism	25	8
Lipid metabolism	11	2
Biosynthesis of secondary metabolites	8	2
Genetic information processing	4	11
Metabolism of cofactors and vitamins	1	7
Other pathways	20	10

Further analysis showed that the most frequently activated disease resistance related pathway by *Mor* in rice was ‘phenylalanine, tyrosine and tryptophan biosynthesis’ (osa00400), followed by ‘phenylalanine metabolism’ (osa00360), ‘phenylpropanoid biosynthesis’ (osa00940), ‘biosynthesis of antibiotics’ (osa01130), and ‘plant-pathogen interaction’ (osa04626) (Table 11). In contrast, ‘plant hormone signal transduction’ (osa04075), and ‘circadian rhythm-plant’ (osa04712) were the most frequently repressed pathways related to disease resistance by *Mor* in rice (Table 11).

**Table 11:** The distribution of the enriched KEGG pathways related to disease resistance of the genes induced by *Mor in the rice microarray data.* Up-Reg: Up-regulation; Down-Reg: Down-regulation

Disease resistance related pathways	Reference	Number of sample	
		Up-Reg	Down-Reg
osa00400:Phenylalanine, tyrosine and tryptophan biosynthesis	[32]	6	0
osa00360:Phenylalanine metabolism	[29–31]	4	0
osa00940:Phenylpropanoid biosynthesis	[25–28]	4	0
osa01130:Biosynthesis of antibiotics		4	1
osa04626:Plant-pathogen interaction		4	1
osa00900:Terpenoid backbone biosynthesis	[46]	3	0
osa03050:Proteasome	[51, 52]	3	0
osa00053:Ascorbate and aldarate metabolism	[53, 54]	2	0
osa00480:Glutathione metabolism	[48]	2	0
osa00945:Stilbenoid, diarylheptanoid and gingerol biosynthesis	[38–40]	2	0
osa00941:Flavonoid biosynthesis	[45]	2	0
osa04075:Plant hormone signal transduction	[21, 23, 33–37]	1	4
osa04144:Endocytosis	[55–57]	1	0
osa04712:Circadian rhythm - plant	[42–44]	0	3
osa03015:mRNA surveillance pathway	[58]	0	2
osa03022:Basal transcription factors		0	2
osa03410:Base excision repair		0	1
osa03430:Mismatch repair		0	1
osa04130:SNARE interactions in vesicular transport	[59, 60]	0	1
osa03450:Non-homologous end-joining	[41]	0	1

## Discussion

Transcriptomic analysis is a powerful tool to reveal the interactions between host and pathogen [61–64]. In this study, we analyzed 35 pairs of samples from 69 pairs of *Xoo*- or *Mor*-infected rice microarray samples deposited in the GEO database. Some rice genes were identified to be frequently regulated by *Xoo* and *Mor* in various conditions. During the interactions between rice and these two pathogens, the pathogen attacks coincided with rice defenses. We separated the up-regulated genes and down-regulated genes by pathogens in rice, in other words, there was no gene overlap between them. GO, InterPro and KEGG annotations and enrichment analyses were performed respectively on the up- and down-regulated genes in each rice sample infected by *Xoo* or *Mor*. We further revealed the frequent GO, InterPro and KEGG annotations and enrichment results of rice genes regulated by *Xoo* and *Mor*.

Some studies have been carried out to investigate the responsive mechanisms in rice to the infections of *Xoo* and *Mor* in specific conditions. The roles of resistance genes were focused on in early studies on rice response to *Xoo* infection [12]. Recently, analyses of transcriptomic data revealed the detailed responses to *Xoo* and *Mor* infections in rice. It has been demonstrated that the transcriptional changes during the compatible interaction between rice and *Mor* were very similar to the changes of incompatible interaction; nevertheless, the changes were higher during the incompatible interaction [8]. In plants including rice, transcriptional reprogramming is a universal process during the infections of pathogens [9, 65, 66]. Our results indicate that the frequently regulated genes involved in basic biological processes from transcription to translation, and their regulation.

A number of researches reported the expression profiles of rice defense response genes [9, 67]. For example, chitinase activities were detected to play roles in rice resistance to *Mor* [68, 69]. Our results show that the expected defense enzymes were the most frequent products of the regulated genes by *Xoo* and *Mor* (Fig. 2, Fig. 5, Additional file 12: Table S9, Additional file 11: S8. Additional file 16: S13. and Additional file 17: S14).

Genes related to secondary metabolism were observed to be up-regulated and significantly enriched in rice infected by *Mor* [8]. In *Arabidopsis*, metabolic pathways were observed to be frequently influenced by diverse pathogens [70]. The resistance to *Mor* was shown in wild rice plants to be involved in lipid metabolism, phenylpropanoid and diterpenoid metabolism [69]. During the interaction of a pair of rice near-isogenic lines (NILs) with *Xoo*, phenylpropanoid biosynthesis was identified as the most conspicuous pathway [11]. In our analysis, KEGG enrichment indicated that ‘phenylpropanoid biosynthesis’ (osa00940) and ‘biosynthesis of secondary metabolites’ (osa01110) were observed to be two of the most frequently enriched pathways in the up-regulated genes by *Xoo* and *Mor* (Fig. 5, Additional file 30: Table S27 and Additional file 32: Table S29). However, ‘biosynthesis of secondary metabolites’ (osa01110) was also the most frequent pathway targeted by the down-regulated genes by *Xoo* (Additional file 31: Table S28). Our analysis further showed ‘biosynthesis of antibiotics’ (osa01130) and ‘biosynthesis of amino acids’ (osa01230) were another two of the pathways most frequently enriched by the up-regulated genes by *Xoo* and *Mor* (Fig. 5, Additional file 30: Table S27 and Additional file 32: Table S29).

Analysis of the DRR/DSR genes responsive to *Xoo* and *Mor* infections suggests that various disease resistance mechanisms were activated or repressed in rice. Among the mechanisms, defense response, signaling pathways, PCD, HR and other cell death, basal and innate immunity were the most eminent arenas that fight between rice and these two pathogens (Fig. 4, Additional file 1: Figure S1 and Additional file 2: Figure S2). It is worth noting that genes encoding antifungal proteins and related to the synthesis of phytoalexin were activated in rice after *Mor* but *Xoo* infection, suggesting that different mechanisms were triggered (Fig. 5 and Additional file 2: Figure S2).

WRKY transcription factors were again and again observed to be implicated in the rice resistance to *Mor* [8, 68, 69]. In our analysis, WRKY proteins (IPR003657) were the most frequently activated transcription factors by *Mor* (Table S26). The WRKY factors were also frequently up-regulated by *Xoo* (Fig. 5, Additional file 8: Table S5). In addition, the zinc finger domain proteins, and the Myb domain proteins were demonstrated to be the most significant factors with differential expressions in most samples infected by *Xoo* and *Mor* (Fig. 5, Additional file 8: Table S5 and Additional file 27: Table S24).

Signalings mediated by abscisic acid and cytokinin were revealed to be successively activated by *Mor* infection in rice [22]. Abscisic acid was also suggested being important in the rice (carrying *Xa7*) response to *Xoo* infection and high temperature stress [10]. However, our results indicate that the processes relative to abscisic acid may not only be frequently activated by the infections of *Xoo* and *Mor* (GO:0009737~response to abscisic acid; GO:0009738~abscisic acid-activated signaling pathway) (Fig. 3 and Fig. 5), but also be repressed by *Mor* infection (GO:0009738~abscisic acid-activated signaling pathway; GO:0009788~negative regulation of abscisic acid-activated signaling pathway) (Fig. 3b). In our results, processes with relation to cytokinin were strikingly and frequently repressed by *Xoo* and *Mor* infections (GO:0009735~response to cytokinin; GO:0009736~cytokinin-activated signaling pathway) (Fig. 3 and Fig. 5). Two pathways mediated by jasmonic acid and ethylene were demonstrated to be activated by *Xoo* and *Mor* infections [11, 68, 69]. Our results show that the processes relative to jasmonic acid were frequently activated in the *Xoo-* and *Mor-*infected rice (GO:2000022~regulation of jasmonic acid mediated signaling pathway; GO:0009695~jasmonic acid biosynthetic process; GO:0009753~response to jasmonic acid) (Fig. 3 and Fig. 5), and they may be repressed by *Mor* infection (Fig. 3b and Fig. 5). The processes relative to ethylene (GO:0009693~ethylene biosynthetic process; GO:0009723~response to ethylene; GO:0009873~ethylene-activated signaling pathway) were also repressed in *Mor-*infected rice (Fig. 3b).

Previous studies have shown that the mechanisms by which rice plants respond to the infections of *Xoo* and *Mor* are not quite identical or even completely opposite under different experimental systems and experimental conditions. Our results reveal which mechanisms are occurring with a relatively high probability.

We show that *Os04g0650800* was most frequently activated by *Xoo.* This gene encodes the D-3-phosphoglycerate dehydrogenase 3 in chloroplast. It has been established that the phosphorylated pathway in plastidial glycolysis is one of three different serine biosynthesis pathways in plants, and 3-phosphoglycerate dehydrogenase (PGDH) is the first committed enzyme [71]. PGDH was revealed to be quantitatively the most important enzyme in maintaining serine homeostasis at the whole plant level [72]. Our result suggests that *Os04g0650800* is likely a key gene in rice defense response and the activation of serine biosynthesis dependent on a phosphorylated pathway may be important in rice response to *Xoo* invasion.

As shown above, the genes responsive to *Xoo* and *Mor* infections may be up-regulated or down-regulated. Further experiments of molecular genetics are necessary to determine that the up- or down-regulated expressions of these genes are active or passive, and to reveal the significance of the genes with highly frequent expressions in response to the infections of these two pathogens. In this study, only six pairs of *Mor*-infected samples were retrieved and analyzed, therefore more extensive analyses are needed on the genes and mechanisms responsive to *Mor* infection in rice.

## Conclusions

A robust set of genes has been defined in rice response to the infections of *Xoo* and *Mor*. Mitochondrion and chloroplast may be important organelles for rice response to *Xoo* and *Mor* infections. Processes with relation to cytokinin, jasmonic acid, and abscisic acid were most frequently operated by *Xoo* and *Mor*. A great number of enzymes were in rice favored to be manipulated by *Xoo* and *Mor*. Defense responses and diverse signaling pathways were indispensable among the responsive mechanisms of rice to *Xoo* and *Mor*. Pathways including phenylpropanoid biosynthesis, biosynthesis of antibiotics, phenylalanine metabolism, and biosynthesis of secondary metabolites were most frequently triggered by *Xoo* and *Mor*. Circadian rhythm-plant was the most frequent pathway repressed by *Xoo* and *Mor*.

## Materials and methods

### Retrieval and analysis of the microarray datasets of rice infected by *Xoo* and *Mor*

The *Xoo-* and *Mor-*infected microarray data were collected from the Gene Expression Omnibus (GEO) database (https://www.ncbi.nlm.nih.gov/geo/) [73, 74]. Sixty-nine pairs (including control and treatment) of raw microarray data (.CEL) of rice in response to the infections of *Xoo* and *Mor* were obtained by SRA Toolkit. The microarray data included 15 series data sets consisting of 51 pairs of *Xoo*-infected rice samples and 18 pairs of *Mor*-infected rice samples. All the microarray experiments were conducted on the same Affymetrix platform (Rice Genome Array GPL2025). The Bioconductor package Simpleaffy was used for quality assessment [75]. GEOquery and limma R packages from GEO2R were used to identify the differential expression of all samples [76]. Poor quality samples were discarded and all the rest of samples (35 pairs of samples) were used to analyze the differentially expressed genes.

### Identification of the differentially expressed genes (DEGs) induced by *Xoo* and *Mor* infections

A series of data generated by GEO2R were then evaluated and analyzed. The obtained *p*-values for multiple testing were corrected according to Benjamini and Hochberg procedure [77]. Probe IDs were converted to gene symbols according to the GPL2025 annotation file provided by GEO. To identify the DEGs, an adjusted *p*-value was used to filter genes with no differential expressions. By setting the threshold of adj. *P*-value < 0.05, the genes with differential expression induced by *Xoo* and *Mor* were screened. Given that more than 30,000 genes were found in rice genome, and more than 1000 DEGs were indentified in many rice samples infected by pathogens, the infected samples with too few DEGs may be resulted from unknown experiment factors. Among the analyzed samples, 34 samples were found with more than 1000 DEGs, and a sample was found with nearly 1000 DEGs (989 DEGs). To avoid inaccurate results, samples with less than 989 DEGs were discarded. In the discarded 34 samples, no DEGs could be identified in 13 samples, DEGs with less than 200 genes were identified from nine samples, and the other samples were found with several hundred DEGs.

### Functional annotation and enrichment analysis for candidate DEGs induced by *Xoo* and *Mor*

The DEGs were annotated through the Rice Genome Annotation Project (MSU, http://rice.plantbiology.msu.edu) [78]. For further InterPro annotation, homology searches were performed against the protein databases (Panther-12.0, PfamA-31.0, PRINTS-42.0, ProDom-2006.1, SuperFamily-1.75, and TIGRFAM15.0), using the local InterProScan program (ver.5.31) [79, 80].

To reveal the potential function tendency of the above identified DEGs, GO enrichment was conducted through the DAVID tool (https://david.ncifcrf.gov/) [81, 82]. And the enrichment analysis was deduced based on *p*-value ≤0.05. Pathway analysis was also carried out through the DAVID tool to unravel the functional annotation of the identified DEGs, and the pathways with *p*-value ≤0.05 were retrieved as significant enrichment [81, 82].

## Supplementary information

**Additional file 1: Figure S1.** The number of unique and common differentially expressed genes (DEGs) present in at least three pairs of rice samples infected by *Xoo* and *Mor*. (A) Up-regulated genes; (B) Down-regulated genes.

**Additional file 2: Figure S2.** Analysis on disease resistance mechanism of the up-regulated DRR/DSR proteins by *Mor* in rice.

**Additional file 3: Figure S3.** Analysis on disease resistance mechanism of the down-regulated DRR/DSR proteins by *Mor* in rice.

**Additional file 4: Table S1.** 69 rice microarray assays used in this study.

**Additional file 5: Table S2.** The number of differentially expressed genes (DEGs) induced by *Xoo* and *Mor.*

**Additional file 6: Table S3.** The DEGs present in at least one sample infected by *Xoo* or *Mor* with pathogen-inducible *cis*-regulatory elements (PICEs) in their promoters.

**Additional file 7: Table S4.** The DEGs present in at least three samples infected by *Xoo* or at least two samples infected by *Mor* with PICEs in their promoters.

**Additional file 8: Table S5.** The distribution of the cellular component ontology for the up-regulated genes by *Xoo* in the rice microarray data.

**Additional file 9: Table S6.** The distribution of the cellular component ontology for the down-regulated genes by *Xoo* in the rice microarray data.

**Additional file 10: Table S7.** The distribution of the biological process ontology for the up-regulated genes by *Xoo* in the rice microarray data.

**Additional file 11: Table S8.** The distribution of the biological process ontology for the down-regulated genes by *Xoo* in the rice microarray data.

**Additional file 12: Table S9.** The distribution of the molecular functions of the up-regulated genes by *Xoo* in the rice microarray data.

**Additional file 13: Table S10.** The distribution of the molecular functions of the down-regulated genes by *Xoo* in the rice microarray data.

**Additional file 14: Table S11.** The distribution of the cellular component ontology for the up-regulated genes by *Mor* in the rice microarray data.

**Additional file 15: Table S12.** The distribution of the cellular component ontology for the down-regulated genes by *Mor* in the rice microarray data.

**Additional file 16: Table S13.** The distribution of the biological process ontology for the up-regulated genes by *Mor* in the rice microarray data.

**Additional file 17: Table S14.** The distribution of the biological process ontology for the down-regulated genes by *Mor* in the rice microarray data.

**Additional file 18: Table S15.** The distribution of the molecular functions of the up-regulated genes by *Mor* in the rice microarray data.

**Additional file 19: Table S16.** The distribution of the molecular functions of the down-regulated genes by *Mor* in the rice microarray data.

**Additional file 20: Table S17.** The distribution of the INTERPRO annotations of the up-regulated genes by *Xoo* in the rice microarray data.

**Additional file 21: Table S18.** The distribution of the INTERPRO annotations of the down-regulated genes by *Xoo* in the rice microarray data.

**Additional file 22: Table S19.** The distribution of the INTERPRO annotations of the up-regulated genes by *Mor* in the rice microarray data.

**Additional file 23: Table S20.** The distribution of the INTERPRO annotations of the down-regulated genes by *Mor* in the rice microarray data.

**Additional file 24: Table S21.** The putative DRRG/DSRGs of the up-regulated genes identified from the rice microarray data infected by *Xoo.*

**Additional file 25: Table S22.** The putative DRRG/DSRGs of the down-regulated genes identified from the rice microarray data infected by *Xoo.*

**Additional file 26: Table S23.** The putative DRRG/DSRGs of the up-regulated genes identified in the rice microarray data infected by *Mor.*

**Additional file 27: Table S24.** The putative DRRG/DSRGs of the down-regulated genes identified in the rice microarray data infected by *Mor.*

**Additional file 28: Table S25.** The putative differentially expressed transcription factors identified in the rice microarray data infected by *Xoo.*

**Additional file 29: Table S26.** The putative differentially expressed transcription factors identified in the rice microarray data infected by *Mor.*

**Additional file 30: Table S27.** The distribution of KEGG annotations of the up-regulated genes by *Xoo* in the rice microarray data.

**Additional file 31: Table S28.** The distribution of KEGG annotations of the down-regulated genes by *Xoo* in the rice microarray data.

**Additional file 32: Table S29.** The distribution of KEGG annotations of the up-regulated genes by *Mor* in the rice microarray data.

**Additional file 33: Table S30.** The distribution of KEGG annotations of the down-regulated genes by *Mor* in the rice microarray data.

## Data Availability

The datasets supporting the conclusions of this article are included within the article and its additional files.

## Abbreviations

ABA: Abscisic acid
DEGs: Differentially expressed genes
DRR/DSR: Disease resistance/susceptibility-related
ET: Ethylene
ETI: Effector-triggered immunity
GEO: Gene Expression Omnibus
GO: Gene Ontology
HR: Hypersensitive reaction
JA: Jasmonic acid
KEGG: Kyoto Encyclopedia of Genes and Genomes
*Mor*: *Magnaporthe oryzae*
PAMP: Pathogen-associated molecular pattern
PCD: Programmed cell death
PICEs: Pathogen-inducible *cis*-regulatory elements
PRRs: Pattern recognition receptors
PTI: PAMP-triggered immunity
ROS: Reactive oxygen species
SA: Salicylic acid
*Xoo*: *Xanthomonas oryzae* pv. *oryzae*

## References

[1] Boller T, He SY (2009). Innate immunity in plants: an arms race between pattern recognition receptors in plants and effectors in microbial pathogens. Science..

[2] Dodds PN, Rathjen JP (2010). Plant immunity: towards an integrated view of plant-pathogen interactions. Nat Rev Genet.

[3] Jones JD, Dangl JL (2006). The plant immune system. Nature..

[4] Chisholm ST, Coaker G, Day B, Staskawicz BJ (2006). Host-microbe interactions: shaping the evolution of the plant immune response. Cell..

[5] Li B, Meng X, Shan L, He P (2016). Transcriptional regulation of pattern-triggered immunity in plants. Cell Host Microbe.

[6] Maekawa T, Kufer TA, Schulze-Lefert P (2011). NLR functions in plant and animal immune systems: so far and yet so close. Nat Immunol.

[7] Gassmann W, Bhattacharjee S (2012). Effector-triggered immunity signaling: from gene-for-gene pathways to protein-protein interaction networks. Mol Plant-Microbe Interact.

[8] Wei T, Ou B, Li J, Zhao Y, Guo D, Zhu Y (2013). Transcriptional profiling of rice early response to *Magnaporthe oryzae* identified *OsWRKYs* as important regulators in rice blast resistance. PLoS One.

[9] Sharma TR, Das A, Thakur S, Devanna BN, Singh PK, Jain P (2016). Oscillating transcriptome during rice-*Magnaporthe* interaction. Curr Issues Mol Biol.

[10] Cohen SP, Liu H, Argueso CT, Pereira A, Vera Cruz C, Verdier V (2017). RNA-Seq analysis reveals insight into enhanced rice *Xa7*-mediated bacterial blight resistance at high temperature. PLoS One.

[11] Tariq Rezwan, Wang Chunlian, Qin Tengfei, Xu Feifei, Tang Yongchao, Gao Ying, Ji Zhiyuan, Zhao Kaijun (2018). Comparative Transcriptome Profiling of Rice Near-Isogenic Line Carrying Xa23 under Infection of Xanthomonas oryzae pv. oryzae. International Journal of Molecular Sciences.

[12] White FF, Yang B (2009). Host and pathogen factors controlling the rice-*Xanthomonas oryzae* interaction. Plant Physiol.

[13] Liu J, Wang X, Mitchell T, Hu Y, Liu X, Dai L (2010). Recent progress and understanding of the molecular mechanisms of the rice-*Magnaporthe oryzae* interaction. Mol Plant Pathol.

[14] Chen X, Ronald PC (2011). Innate immunity in rice. Trends Plant Sci.

[15] Shen X, Yuan B, Liu H, Li X, Xu C, Wang S (2010). Opposite functions of a rice mitogen-activated protein kinase during the process of resistance against *Xanthomonas oryzae*. Plant J.

[16] Shen X, Liu H, Yuan B, Li X, Xu C, Wang S (2011). *OsEDR1* negatively regulates rice bacterial resistance via activation of ethylene biosynthesis. Plant Cell Environ.

[17] Deng H, Liu H, Li X, Xiao J, Wang S (2012). A CCCH-type zinc finger nucleic acid-binding protein quantitatively confers resistance against rice bacterial blight disease. Plant Physiol.

[18] Yamada S, Kano A, Tamaoki D, Miyamoto A, Shishido H, Miyoshi S (2012). Involvement of *OsJAZ8* in jasmonate-induced resistance to bacterial blight in rice. Plant Cell Physiol.

[19] Xu J, Audenaert K, Hofte M, De Vleesschauwer D (2013). Abscisic acid promotes susceptibility to the rice leaf blight pathogen *Xanthomonas oryzae* pv. *oryzae* by suppressing salicylic acid-mediated defenses. PLoS One.

[20] Xu J, Zhou L, Venturi V, He YW, Kojima M, Sakakibari H (2015). Phytohormone-mediated interkingdom signaling shapes the outcome of rice-*Xanthomonas oryzae* pv *oryzae* interactions. BMC Plant Biol.

[21] Yang DL, Yang Y, He Z (2013). Roles of plant hormones and their interplay in rice immunity. Mol Plant.

[22] Cao J, Yang C, Li L, Jiang L, Wu Y, Wu C (2016). Rice plasma membrane proteomics reveals *Magnaporthe oryzae* promotes susceptibility by sequential activation of host hormone signaling pathways. Mol Plant-Microbe Interact.

[23] Muller M, Munne-Bosch S (2015). Ethylene response factors: a key regulatory hub in hormone and stress signaling. Plant Physiol.

[24] Kong W, Ding L, Cheng J, Wang B (2018). Identification and expression analysis of genes with pathogen-inducible cis-regulatory elements in the promoter regions in *Oryza sativa*. Rice..

[25] Franke R, Hemm MR, Denault JW, Ruegger MO, Humphreys JM, Chapple C (2002). Changes in secondary metabolism and deposition of an unusual lignin in the *ref8* mutant of *Arabidopsis*. Plant J.

[26] Shadle GL, Wesley SV, Korth KL, Chen F, Lamb C, Dixon RA (2003). Phenylpropanoid compounds and disease resistance in transgenic tobacco with altered expression of L-phenylalanine ammonia-lyase. Phytochemistry..

[27] El-kereamy A, El-sharkawy I, Ramamoorthy R, Taheri A, Errampalli D, Kumar P (2011). *Prunus domestica* pathogenesis-related protein-5 activates the defense response pathway and enhances the resistance to fungal infection. PLoS One.

[28] Cass CL, Peraldi A, Dowd PF, Mottiar Y, Santoro N, Karlen SD (2015). Effects of PHENYLALANINE AMMONIA LYASE (PAL) knockdown on cell wall composition, biomass digestibility, and biotic and abiotic stress responses in *Brachypodium*. J Exp Bot.

[29] Shine MB, Yang JW, El-Habbak M, Nagyabhyru P, Fu DQ, Navarre D (2016). Cooperative functioning between phenylalanine ammonia lyase and isochorismate synthase activities contributes to salicylic acid biosynthesis in soybean. New Phytol.

[30] Kim DS, Hwang BK (2014). An important role of the pepper phenylalanine ammonia-lyase gene (PAL1) in salicylic acid-dependent signalling of the defence response to microbial pathogens. J Exp Bot.

[31] Chezem WR, Memon A, Li FS, Weng JK, Clay NK (2017). SG2-type R2R3-MYB transcription factor MYB15 controls defense-induced lignification and basal immunity in *Arabidopsis*. Plant Cell.

[32] Powell JJ, Carere J, Fitzgerald TL, Stiller J, Covarelli L, Xu Q (2017). The *Fusarium* crown rot pathogen *Fusarium pseudograminearum* triggers a suite of transcriptional and metabolic changes in bread wheat (*Triticum aestivum* L.). Ann Bot.

[33] Llorente F, Muskett P, Sanchez-Vallet A, Lopez G, Ramos B, Sanchez-Rodriguez C (2008). Repression of the auxin response pathway increases *Arabidopsis* susceptibility to necrotrophic fungi. Mol Plant.

[34] Fan J, Hill L, Crooks C, Doerner P, Lamb C (2009). Abscisic acid has a key role in modulating diverse plant-pathogen interactions. Plant Physiol.

[35] Reusche M, Klaskova J, Thole K, Truskina J, Novak O, Janz D (2013). Stabilization of cytokinin levels enhances *Arabidopsis* resistance against *Verticillium longisporum*. Mol Plant-Microbe Interact.

[36] Shi Hua, Yan Haojie, Li Juan, Tang Dingzhong (2013). BSK1, a receptor-like cytoplasmic kinase, involved in both BR signaling and innate immunity inArabidopsis. Plant Signaling & Behavior.

[37] Wang S, Wang S, Sun Q, Yang L, Zhu Y, Yuan Y (2017). A role of cytokinin transporter in *Arabidopsis* immunity. Mol Plant-Microbe Interact.

[38] Huang L, Zhang S, Singer SD, Yin X, Yang J, Wang Y (2016). Expression of the grape *Vqsts21* gene in *Arabidopsis* confers resistance to osmotic stress and biotrophic pathogens but not *Botrytis cinerea*. Front Plant Sci.

[39] Zeng W, Sun Z, Cai Z, Chen H, Lai Z, Yang S (2017). Proteomic analysis by iTRAQ-MRM of soybean resistance to *Lamprosema Indicate*. BMC Genomics.

[40] Xu J, Li M, Jiao P, Tao H, Wei N, Ma F (2015). Dynamic transcription profiles of "Qinguan" apple (*Malus* x domestica) leaves in response to *Marssonina coronaria* inoculation. Front Plant Sci.

[41] Richter KS, Jeske H (2015). KU80, a key factor for non-homologous end-joining, retards geminivirus multiplication. J Gen Virol.

[42] Sauerbrunn N, Schlaich NL (2004). PCC1: a merging point for pathogen defence and circadian signalling in *Arabidopsis*. Planta..

[43] Griebel T, Zeier J (2008). Light regulation and daytime dependency of inducible plant defenses in *Arabidopsis*: phytochrome signaling controls systemic acquired resistance rather than local defense. Plant Physiol.

[44] Fu S, Shao J, Zhou C, Hartung JS (2016). Transcriptome analysis of sweet orange trees infected with '*Candidatus Liberibacter* asiaticus' and two strains of Citrus Tristeza virus. BMC Genomics.

[45] Pasold S, Siegel I, Seidel C, Ludwig-Muller J (2010). Flavonoid accumulation in *Arabidopsis thaliana* root galls caused by the obligate biotrophic pathogen *Plasmodiophora brassicae*. Mol Plant Pathol.

[46] Lange BM (2015). The evolution of plant secretory structures and emergence of terpenoid chemical diversity. Annu Rev Plant Biol.

[47] Testone G, Bruno L, Condello E, Chiappetta A, Bruno A, Mele G (2008). Peach [*Prunus persica* (L.) Batsch] KNOPE1, a class 1 KNOX orthologue to *Arabidopsis* BREVIPEDICELLUS/KNAT1, is misexpressed during hyperplasia of leaf curl disease. J Exp Bot.

[48] Parisy V, Poinssot B, Owsianowski L, Buchala A, Glazebrook J, Mauch F (2007). Identification of PAD2 as a gamma-glutamylcysteine synthetase highlights the importance of glutathione in disease resistance of *Arabidopsis*. Plant J.

[49] Yin L, Qu J, Deng S, Liu S, Lu J, Zhang Y (2017). Phytohormone and genome variations in *Vitis amurensis* resistant to downy mildew. Genome..

[50] Chen Y, Dong J, Bennetzen JL, Zhong M, Yang J, Zhang J (2017). Integrating transcriptome and microRNA analysis identifies genes and microRNAs for AHO-induced systemic acquired resistance in *N. tabacum*. Sci Rep..

[51] Copeland C, Woloshen V, Huang Y, Li X (2016). AtCDC48A is involved in the turnover of an NLR immune receptor. Plant J.

[52] Yao C, Wu Y, Nie H, Tang D (2012). RPN1a, a 26S proteasome subunit, is required for innate immunity in *Arabidopsis*. Plant J.

[53] Grosskinsky DK, Koffler BE, Roitsch T, Maier R, Zechmann B (2012). Compartment-specific antioxidative defense in *Arabidopsis* against virulent and avirulent *Pseudomonas syringae*. Phytopathology..

[54] Botanga CJ, Bethke G, Chen Z, Gallie DR, Fiehn O, Glazebrook J (2012). Metabolite profiling of *Arabidopsis* inoculated with *Alternaria brassicicola* reveals that ascorbate reduces disease severity. Mol Plant-Microbe Interact.

[55] Hatsugai N, Hillmer R, Yamaoka S, Hara-Nishimura I, Katagiri F (2016). The mu subunit of *Arabidopsis* adaptor protein-2 is involved in effector-triggered immunity mediated by membrane-localized resistance proteins. Mol Plant-Microbe Interact.

[56] Kawchuk LM, Hachey J, Lynch DR, Kulcsar F, van Rooijen G, Waterer DR (2001). Tomato *Ve* disease resistance genes encode cell surface-like receptors. Proc Natl Acad Sci U S A.

[57] Ron M, Avni A (2004). The receptor for the fungal elicitor ethylene-inducing xylanase is a member of a resistance-like gene family in tomato. Plant Cell.

[58] Gloggnitzer J, Akimcheva S, Srinivasan A, Kusenda B, Riehs N, Stampfl H (2014). Nonsense-mediated mRNA decay modulates immune receptor levels to regulate plant antibacterial defense. Cell Host Microbe.

[59] Sugano S, Hayashi N, Kawagoe Y, Mochizuki S, Inoue H, Mori M (2016). Rice OsVAMP714, a membrane-trafficking protein localized to the chloroplast and vacuolar membrane, is involved in resistance to rice blast disease. Plant Mol Biol.

[60] Acevedo-Garcia J, Collins NC, Ahmadinejad N, Ma L, Houben A, Bednarek P (2013). Fine mapping and chromosome walking towards the *Ror1* locus in barley (*Hordeum vulgare* L.). Theor Appl Genet.

[61] Becker MG, Haddadi P, Wan J, Adam L, Walker P, Larkan NJ (2019). Transcriptome analysis of *Rlm2*-mediated host immunity in the *Brassica napus*–*Leptosphaeria maculans* pathosystem. Mol Plant-Microbe Interact.

[62] Karmakar K, Kundu A, Rizvi AZ, Dubois E, Severac D, Czernic P (2019). Transcriptomic analysis with the progress of symbiosis in ‘crack-entry’ legume *Arachis hypogaea* highlights its contrast with ‘infection thread’ adapted legumes. Mol Plant-Microbe Interact.

[63] Yu D, Fang Y, Tang C, Klosterman SJ, Tian C, Wang Y (2019). Genomewide transcriptome profiles reveal how bacillus subtilis lipopeptides inhibit microsclerotia formation in *Verticillium dahliae*. Mol Plant-Microbe Interact.

[64] Zhao C, Wang H, Lu Y, Hu J, Qu L, Li Z (2019). Deep sequencing reveals early reprogramming of *Arabidopsis* root transcriptomes upon *Ralstonia solanacearum* infection. Mol Plant-Microbe Interact.

[65] Katagiri F (2004). A global view of defense gene expression regulation--a highly interconnected signaling network. Curr Opin Plant Biol.

[66] Eulgem T (2005). Regulation of the *Arabidopsis* defense transcriptome. Trends Plant Sci.

[67] Vergne E, Ballini E, Marques S, Sidi Mammar B, Droc G, Gaillard S (2007). Early and specific gene expression triggered by rice resistance gene *Pi33* in response to infection by ACE1 avirulent blast fungus. New Phytol.

[68] Jain P, Singh PK, Kapoor R, Khanna A, Solanke AU, Krishnan SG (2017). Understanding host-pathogen interactions with expression profiling of NILs carrying rice-blast resistance *Pi9* gene. Front Plant Sci.

[69] Tian L, Shi S, Nasir F, Chang C, Li W, Tran LP (2018). Comparative analysis of the root transcriptomes of cultivated and wild rice varieties in response to *Magnaporthe oryzae* infection revealed both common and species-specific pathogen responses. Rice..

[70] Jiang Z, He F, Zhang Z (2017). Large-scale transcriptome analysis reveals *Arabidopsis* metabolic pathways are frequently influenced by different pathogens. Plant Mol Biol.

[71] Cascales-Minana B, Munoz-Bertomeu J, Flores-Tornero M, Anoman AD, Pertusa J, Alaiz M (2013). The phosphorylated pathway of serine biosynthesis is essential both for male gametophyte and embryo development and for root growth in *Arabidopsis*. Plant Cell.

[72] Toujani W, Munoz-Bertomeu J, Flores-Tornero M, Rosa-Tellez S, Anoman AD, Alseekh S (2013). Functional characterization of the plastidial 3-phosphoglycerate dehydrogenase family in *Arabidopsis*. Plant Physiol.

[73] Edgar R, Domrachev M, Lash AE (2002). Gene expression omnibus: NCBI gene expression and hybridization array data repository. Nucleic Acids Res.

[74] Barrett T, Wilhite SE, Ledoux P, Evangelista C, Kim IF, Tomashevsky M (2013). NCBI GEO: archive for functional genomics data sets--update. Nucleic Acids Res.

[75] Wilson CL, Miller CJ (2005). Simpleaffy: a BioConductor package for Affymetrix quality control and data analysis. Bioinformatics..

[76] Sean D, Meltzer PS (2007). GEOquery: a bridge between the gene expression omnibus (GEO) and BioConductor. Bioinformatics..

[77] Benjamini Y, Hochberg Y (1995). Controlling the false discovery rate: a practical and powerful approach to multiple testing. J R Stat Soc Ser B Methodol.

[78] Kawahara Y, de la Bastide M, Hamilton JP, Kanamori H, McCombie WR, Ouyang S (2013). Improvement of the *Oryza sativa* Nipponbare reference genome using next generation sequence and optical map data. Rice..

[79] Jones P, Binns D, Chang H-Y, Fraser M, Li W, McAnulla C (2014). InterProScan 5: genome-scale protein function classification. Bioinformatics..

[80] Finn RD, Attwood TK, Babbitt PC, Bateman A, Bork P, Bridge AJ (2017). InterPro in 2017—beyond protein family and domain annotations. Nucleic Acids Res.

[81] Huang DW, Sherman BT, Lempicki RA (2009). Bioinformatics enrichment tools: paths toward the comprehensive functional analysis of large gene lists. Nucleic Acids Res.

[82] Huang DW, Sherman BT, Lempicki RA (2009). Systematic and integrative analysis of large gene lists using DAVID bioinformatics resources. Nat Protoc.

