# Supplementary material for: Identification and characterization of genes frequently responsive to Xanthomonas oryzae pv. oryzae and Magnaporthe oryzae infections in rice
Source: BMC Genomics. 2020 Jan 6;21:21. doi: 10.1186/s12864-019-6438-y (PMC6945429; doi:10.1186/s12864-019-6438-y)
Supplement: Supplementary file 1 — Additional file 1: Figure S1. The number of unique and common differentially expressed genes (DEGs) present in at least three pairs of rice samples infected by Xoo and Mor. (A) Up-regulated genes; (B) Down-regulated genes. [file 12864_2019_6438_MOESM1_ESM.docx]

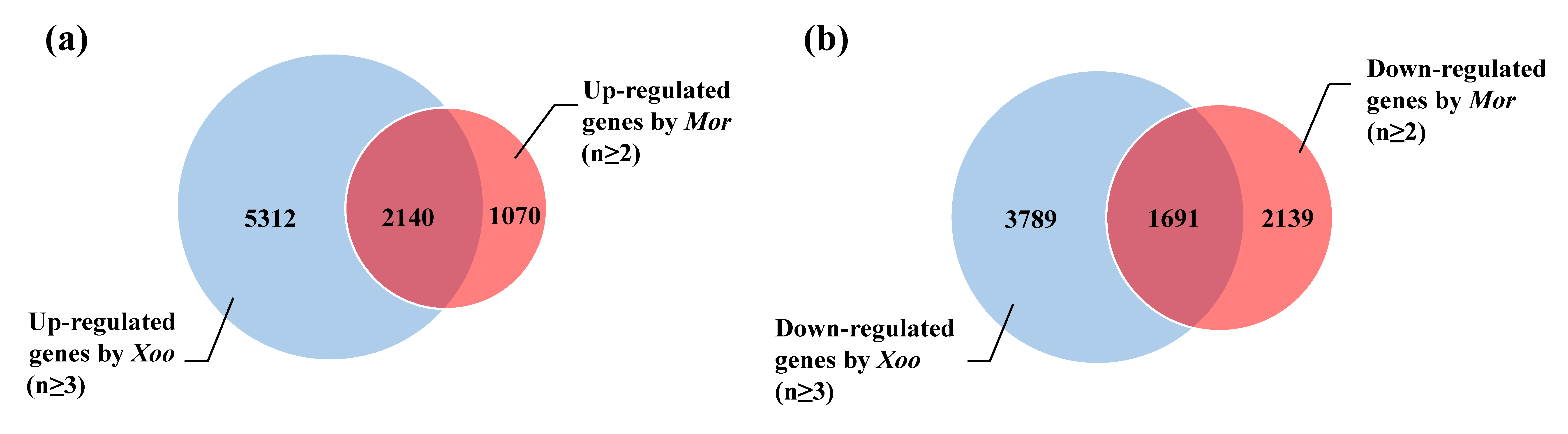


**Figure S1**

**Number of unique and common differentially expressed genes (DEGs) present in at least three rice samples infected by *Xoo* and *Mor*.** (a) Up-regulated genes; (b) Down-regulated genes.
