# Supplementary material for: Identification and characterization of genes frequently responsive to Xanthomonas oryzae pv. oryzae and Magnaporthe oryzae infections in rice
Source: BMC Genomics. 2020 Jan 6;21:21. doi: 10.1186/s12864-019-6438-y (PMC6945429; doi:10.1186/s12864-019-6438-y)
Supplement: Supplementary file 3 — Additional file 3: Figure S3. Analysis on disease resistance mechanism of the down-regulated DRR/DSR proteins by Mor in rice. [file 12864_2019_6438_MOESM3_ESM.docx]

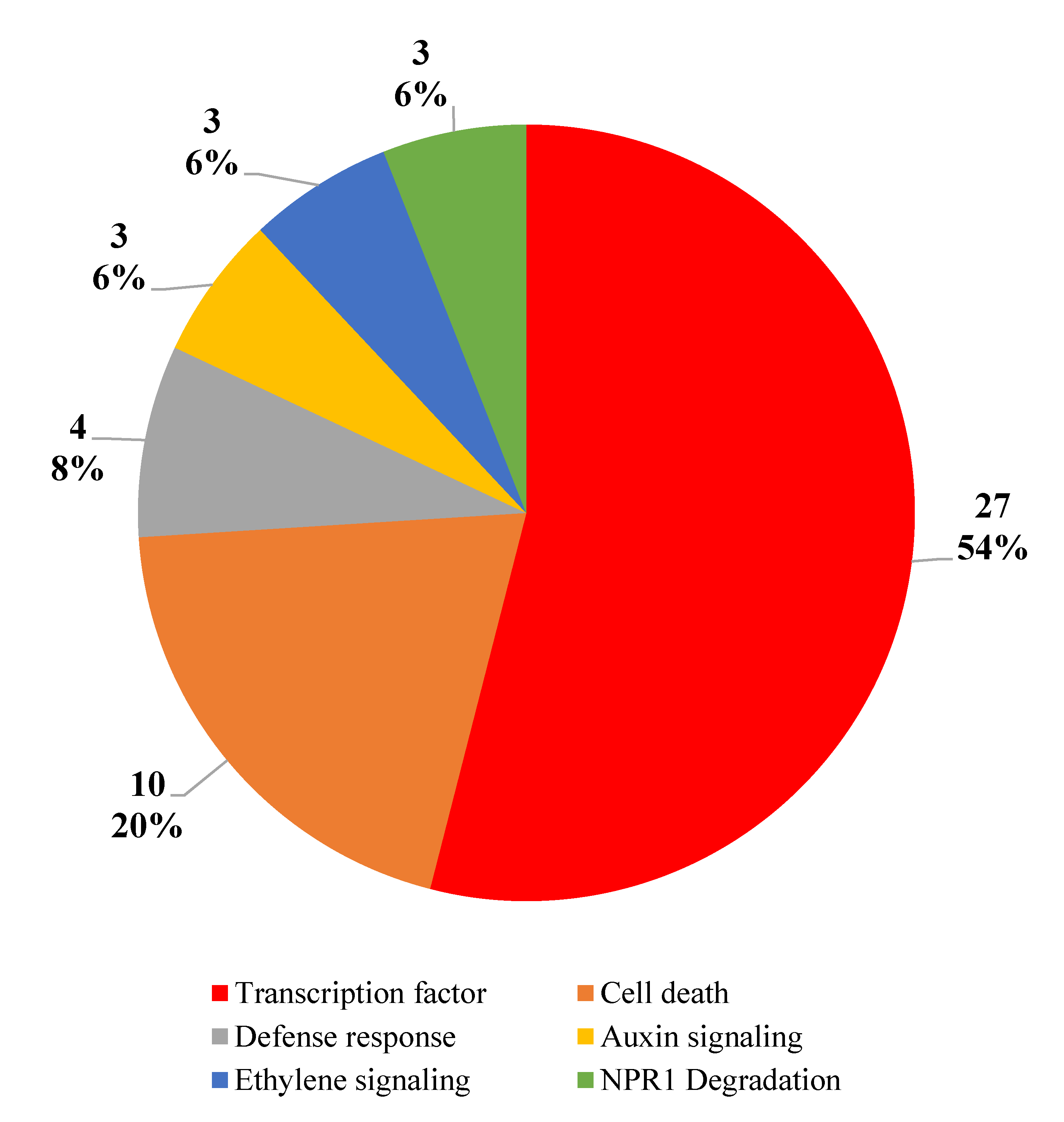


**Figure S3**

**Analysis on disease resistance mechanisms of the down-regulated DRR/DSR proteins by *Mor* in rice**
